# Neural and Behavioral Tracking of Musical Phrases Occurs Without Temporal Regularity

**DOI:** 10.1111/ejn.70481

**Published:** 2026-04-12

**Authors:** Zofia Anna Hołubowska, Xiangbin Teng, Pauline Larrouy‐Maestri

**Affiliations:** ^1^ Max‐Planck‐Institute for Empirical Aesthetics Frankfurt Germany; ^2^ Institute of Applied Psychology, Faculty of Management and Social Communication Jagiellonian University Kraków Poland; ^3^ Max‐Planck‐Institute for Human Cognitive and Brain Sciences Leipzig Germany; ^4^ Institute of Biology, Faculty of Life Sciences University of Leipzig Leipzig Germany; ^5^ Department of Psychology Chinese University of Hong Kong Shatin New Territories, Hong Kong SAR China; ^6^ Brain and Mind Institute The Chinese University of Hong Kong Shatin New Territories, Hong Kong SAR China

**Keywords:** EEG, music perception, music structure, neural tracking, phrase segmentation

## Abstract

When listening to music or speech, people naturally divide continuous sound streams into segments for easier and faster processing of information. The segmentation boundaries are not random. Listeners agree on the points of segmentation, which are consistent with arbitrary rules—for example, those established by music theory—and often occur at regular time intervals. It is thus unclear whether phrase tracking relies on understanding of musical structure or merely on temporal predictability. To address this, we examined how non‐musicians process both regular (temporally predictable) and irregular musical phrases derived from J.S. Bach's compositions. This approach preserved authentic musical structure while manipulating temporal predictability. We also included control stimuli matched in acoustic properties but lacking musical structure. Behavioral and EEG measures revealed that listeners could accurately detect phrase boundaries in both regular and irregular conditions. Neural activity, indexed by an increase in low‐frequency EEG power, reflected the tracking structural boundaries regardless of temporal regularity. These findings demonstrate that musical segmentation depends fundamentally on implicit understanding of musical structure, rather than on temporal predictability alone.

## Graphical abstract

When listening to music or speech, people naturally segment continuous sound streams into meaningful units. We investigated how the temporal predictability of segment boundaries influences listeners' ability to track musical structure. Both neural and behavioral measures showed robust tracking of musical phrasing regardless of boundary regularity, indicating that segmentation relies on implicit structural knowledge rather than solely on temporal predictability.

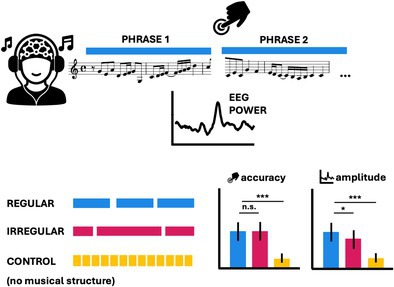

**Abbreviations**
Cacoh: cerebral–acoustic coherence
CSP: closure positive shift
EEG: electroencephalography
ERAN: early right anterior negativity
ERP: event‐related potential
fMRI: functional magnetic resonance imaging
Gold‐MSI: Goldsmiths Music Sophistication Index
ICA: independent component analysis
MCCA: multivariate canonical correlation analysis
PCA: principal component analysis
RMS: root mean square
TRF: temporal response function

## 1 Introduction

The perception of a continuous sensory stream, like speech and music, requires its segmentation into meaningful events. This allows us to make predictions about the near future, update information, and form memories (Kurby and Zacks 2008). Defining boundaries between events is not random. People consistently agree on when an event starts or ends; for example, while reading (Sridharan et al. 2007), watching movies (Swallow and Zacks 2004), or when looking at picture stories (Gernsbacher 1985).

In music, segmentation occurs when a continuous melody is divided into phrases. A phrase could be defined as a series of notes that carries a complete musical sense and forms a natural division in a melodic line (Apel 1969; Nattiez 1990; Rutherford‐Johnson 2013). Phrase segmentation is determined by culturally specific cues, such as those formalized in the Generative Theory of Tonal Music (Lerdahl and Jackendoff 1996). These cues include local changes, such as longer notes signalling the end of a phrase, or even a break (silence) preceding a new phrase (Knösche et al. 2005; Riemann 1900), a change in timbre, sound intensity, or register (Deliège 1987). Global cues arise from harmonic progression, where phrases create predictable harmonic patterns that make phrase boundaries easier to anticipate (Beach 1995; Lerdahl and Jackendoff 1996; Tan et al. 1981). Deliège (1987) has shown that listeners are able to follow those rules and parse music according to the theory, creating defined musical units—phrases.

Multiple studies have confirmed that listeners are able to segment music into phrases using different methods. Behaviorally, participants could be asked to rate perceived completeness of presented sequences of music (Hansen et al. 2021; Palmer and Krumhansl 1987), or to link parts of phrases in order to create a coherent melody (Tillmann et al. 1998). Music segmentation has also been studied with fMRI. Sridharan et al. (2007) found that prominent musical boundaries, such as transitions between musical movements, activate distinct neural networks: an early response in a ventral fronto‐temporal network followed by activation in a dorsal fronto‐parietal network. More recently, Burunat et al. (2024) examined finer‐grained segments in music, identifying two networks: the Early Auditory Network (posterior auditory areas) and the Boundary Transition Network (middle and anterior auditory areas) that process phrase boundaries.

EEG studies have identified several neural markers of phrase segmentation in music. Knösche et al. (2005) demonstrated that music elicits brain responses comparable to the closure positive shift (CPS), an ERP component indicating phrase boundary perception in speech (Steinhauer et al. 1999). Deviating from the syntactic expectations in music is indexed with the early right anterior negativity (ERAN) (Koelsch et al. 2005). In recent study, Teng et al. (2024) introduced a new approach of EEG data analysis and observed the online tracking of structure in music. They used four‐part chorales composed by Johann Sebastian Bach, presented in a passive listening task to describe a new neural marker for phrase tracking: EEG power modulation near phrase boundaries, characterized by an increase in EEG power within the frequency range corresponding to the beat rate in music (~1 Hz). EEG power modulation is a continuous and more direct measure of brain tracking of musical structure than previously used measures, such as ERAN or CPS, which require comparisons between conditions; for example, in the paradigm of expectation violation (Knösche et al. 2005; Koelsch et al. 2000).

Most studies on phrase perception have used highly ordered musical material: simple melodies (Glushko et al. 2016; Knösche et al. 2005; Quiroga‐Martinez et al. 2020), nursery rhymes (Zhang et al. 2016), or easy pieces of classical music such as minuets or chorales (Deliège 1987; Hansen et al. 2021; Teng et al. 2024; Tillmann et al. 1998). These stimuli are typically characterized by phrases of equal length embedded within continuous melodies, meaning that every phrase has the same duration. Such temporal regularity introduces an additional cue that may facilitate the recognition of structural patterns, even without detailed knowledge of the musical cues themselves. Although regular phrase lengths are common in music, they are not universal (Lissa 1987; Temperley and Clercq 2013). This leaves open the question of whether temporal regularity is necessary for building structural predictions. Understanding the role of temporal regularity is important because it serves as a strong cue for auditory stream segregation (Andreou et al. 2011; Large and Palmer 2002). Research on speech processing demonstrates that cortical entrainment, synchronization between neural and acoustic signals, occurs in response to regularly occurring syntactic structures (Ding et al. 2016). Moreover, delta‐band oscillations, which seem to be linked with the syntactic tracking in speech, can bias speech segmentation decisions: when sentences are ambiguous or lack prosodic cues, listeners tend to segment the signal in temporally regular ways, suggesting that we build temporal expectations about unit length. The temporal regularity of phrases is an important cue for music segmentation (Deliège and El Ahnmadi 1990; Nattiez 1990; Zhang et al. 2016). Nevertheless, temporal regularity alone does not capture the essence of what constitutes a musical phrase.

In this study, we examined how temporal regularity (i.e., the predictability of phrase boundary timing) affects listeners' ability to parse music accurately. Temporal regularity arises when phrase boundaries occur at consistent intervals. To test its influence on the segmentation of music, we constructed stimuli based on fugue themes by Johann Sebastian Bach, which naturally vary in phrase length. By combining these themes, we generated longer melodies that preserved the musical cues signalling phrase boundaries while differing in temporal regularity. This allows for better understanding of the contribution of regularity to music segmentation. We contrasted an irregular condition, in which phrases retained their original varying lengths, with a regular condition in which all selected phrases consisted of eight beats, with a boundary every 6.5 s. Thus, although both conditions contained identical boundary cues, only the regular condition showed a predictable temporal pattern of musical structure.

We also included a shuffled control condition in which notes from the original melodies were randomly reordered to remove larger‐scale musical structure.

We assessed the sensitivity of participants to phrase boundaries using both implicit and explicit measures. Implicit tracking was captured through EEG recordings during passive listening, whereas explicit detection was evaluated in a behavioral task in which listeners indicated when they perceived a phrase boundary. This design allowed us to compare how strongly the ability to track musical structure (phrases) depends on the temporal predictability of boundary timing, and how much can be explained with listeners' understanding of the subtle musical cues.

## 2 Methods

### 2.1 Participants

Thirty‐five participants (mean age 26.17 ± 4.11, 25 self‐reported as female, 8 as male, and 2 as other) were recruited from the database of the Max Planck Institute for Empirical Aesthetics. The sample size was determined based on a previous study with comparable experimental design and analysis approach (Teng et al. 2024). A power analysis conducted post hoc in G*Power (Faul et al. 2007), based on the reported effect size in the reference paper ($\eta_{p}^{2}=0.170,{\text{Cohen}}^{'}s\;f=0.45$), indicated that a minimum of 15 participants would be sufficient to detect the effect with 80% power at an alpha level of 0.05.

Participants did not report hearing impairments and showed a rather low level of music expertise. Indeed, the mean General Index of the Goldsmiths Music Sophistication Index (Gold‐MSI) in our sample was 72.00 ± 12.80 on a scale from 18 to 126 (Müllensiefen et al. 2014). Similarly, the mean Musical Training score in our sample was 21.82 ± 8.89 on a scale from 7 to 49. According to the Short Test of Music Preferences questionnaire (STOMP‐R, German adaptation) (Fricke and Herzberg 2017; Rentfrow et al. 2011), participants did not have a significant inclination towards classical music, as it was ranked as the fifth most preferred genre, after pop, soul, rap, and alternative. The experimental procedure followed guidelines ethically approved by the Max Planck Society Ethics Council protocol (application 2018–38) and was compensated with 7€ for every 30 min of their participation.

### 2.2 Material

**FIGURE 1:**
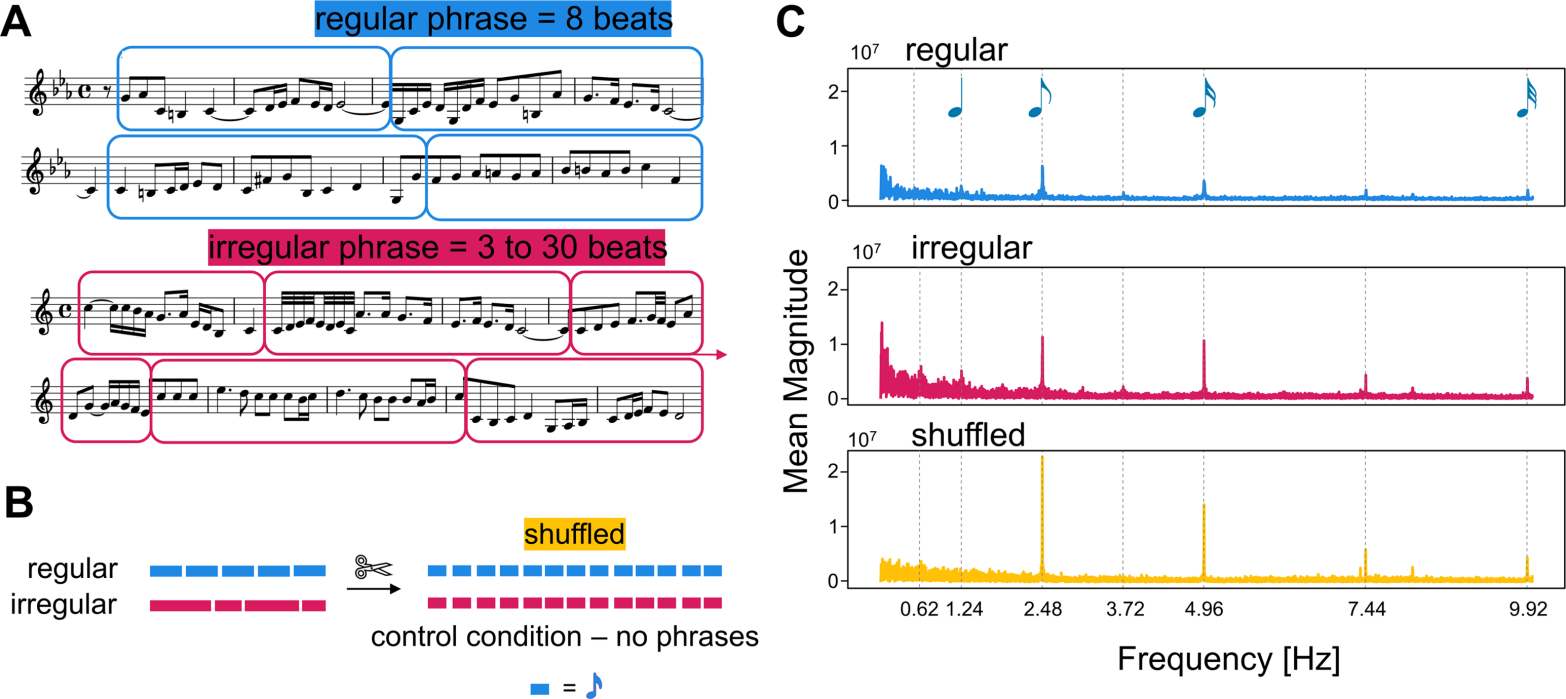
Visualization of a method of creating the stimuli for the experiment. (**A**) Difference in phrasal structure between regular and irregular condition is reflected in the length of phrases present in the musical material. (**B**) A schematic representation on creating the shuffled condition. (**C**) Spectral analysis of the frequencies present in the acoustic envelopes of the stimuli. The results were obtained by applying fast Fourier transform to the acoustic envelope of each of the melodies used as stimuli. Grey lines indicate musically interpretable frequencies.

The goal of creating the music material was twofold: We wanted to obtain maximal ecological validity while varying the length of phrases. We used themes extracted from fugues composed by Johann Sebastian Bach (1685–1750). The full list of compositions could be found in Supporting Information S1: Section S1. The themes of fugues can be described as defined, unified melodies, with strong beginning, which facilitates the recognition of the theme (Harrison 2008), and great variability in length (from 3 to 30 beats per phrase, 9.5 beats on average for our selection of phrases). The process of preparing the stimuli was as follows: themes were isolated from fugues, while keeping their length as in the original pieces. The length was then quantified in number of beats. The themes were transposed to either C major or c minor, maintaining their original mode (i.e., major themes remained in major key, minor themes in minor key). Themes were then categorized according to two factors: mode (major or minor) and phrase regularity (themes with 4, 8, or 12 beats were classified as regular, whereas all others were classified as irregular). This categorization resulted in four sets of phrases (regular‐major, regular‐minor, irregular‐major, irregular‐minor), which were combined to create eight longer melodies ranging from 69 to 233 s (on average 144.13 s). See Figure 1A for examples of regular and irregular melodies with marked phrases, and Supporting Information S1: Section S2 for detailed descriptions of the structure of each stimulus. Each theme appeared twice across the stimuli, but only within different melodies of the same regularity (regular or irregular) and mode (major or minor) condition. Also, following the exploration of the stimulus preparation and listeners' ability to follow the intended structure of melodies (N_pilot_ = 8) we decided to slightly change the ending of phrases by adding some salient cues (enlarged intervals, longer note duration) (Deliège 1987), however no silence in between phrases was introduced. At the same time, we kept the material very close to the original, meaning that longer note durations and bigger interval jumps were present not only at the end of phrases; thus, the correct recognition of a phrase boundary required integration of these cues with the harmonic structure of the phrase. More details on creating the stimuli are provided in the Supporting Information S1: Section S3.

### 2.3 Stimuli

We quantified the relationship between acoustic features and phrase boundaries, by fitting a generalized linear mixed model with a binomial distribution, predicting boundary occurrence from previous note duration, frequency interval, regularity condition (regular vs. irregular), and their two‐way interactions. Both continuous predictors (previous note duration and frequency interval) were standardized (*z*‐scored) prior to analysis to facilitate interpretation and comparison of effect sizes. The model included individual melodies as a random intercept to account for potential melody‐specific variation, though the estimated random effect variance was zero, indicating negligible between‐melody variation beyond that explained by the fixed effects. The model was fitted using maximum likelihood estimation, implemented via the *glmer* function in the *lme4* package in R (Bates et al. 2015).

This analysis revealed that both previous note duration (OR = 2.36, 95% CI [2.00, 2.78], *z* = 10.17, *p* < 0.001) and frequency interval (OR = 3.10, 95% CI [2.56, 3.77], *z* = 11.45, *p* < 0.001) significantly increased the likelihood of phrase boundaries. The main effect of condition was not significant (OR = 1.06, 95% CI [0.50, 2.26], *z* = 0.15, *p* = 0.882), suggesting that phrase boundaries were not systematically more or less likely in the regular condition overall when controlling for acoustic features.

The model explained a substantial amount of variance in boundary occurrence (marginal *R* ^2^ = 0.437). Given that local acoustic changes substantially predicted boundary occurrence, we included these naturally appearing acoustic factors (i.e., previous note duration and frequency interval) as covariates in further analyses.

To create stimuli preserving the acoustic qualities of melodies but with a disrupted harmonic structure, melodies were chunked into half‐beat long units, which were shuffled and assembled to create quasi‐melodies (Figure 1B). As a consequence, the low‐level features (e.g., pitch range, timbre, and loudness) were preserved, but the music structure (phrases) was disrupted.

The audio files were created in MuseScore (Version 3.6.2) with a MIDI piano timbre, at a tempo of 74.4 beats per minute (bpm). This moderate tempo has been chosen within the range of performance tempi suggested in editions of Bach's fugues (Czerny 1906) and allowed to keep the large range of rhythmical content of the phrases used in the stimuli (i.e., containing both whole notes and semiquavers).

To present the auditory stimuli, in‐ear headphones with foam ear tips (ER‐3C Tubal Insert Earphones, 50 Ohm by Etymotic Research) were used. The volume of the stimuli was adjusted to approximately 70 dB using the RME Fireface UC(X) external soundcard with TotalMix FM interface (RME Intelligent Audio Solutions). The volume calibration to 70 dB was performed using a pure 440 Hz tone and the RS PRO SPL‐metre (PR‐1150, RS Components GmbH).

### 2.4 Procedure

After signing a written consent, participants completed the Gold‐MSI and STOMP‐R questionnaires. Then, an EEG session was proposed in a sound‐proof booth. Participants were asked to listen to music while focusing their gaze on a yellow cross displayed on a computer screen. Twelve melodies were played two times, in random order, in six blocks (four melodies each). To maintain participants' attention, they were asked to answer the question “Did it sound like an existing piece of music to you?” on a scale from 1 (*not at all*) to 6 (*very much*) after each melody. After each block, participants were allowed to take a short break.

The last part of the experiment consisted of the behavioral task. EEG data were not recorded for this part of the experiment. Participants were provided with a definition of phrase (*A phrase is a series of notes that displays a complete musical sense and that forms a natural division of the melodic line, constituting a complete whole*.; adapted from the Larousse Encyclopédie from 1957, as cited in Nattiez 1990), accompanied with examples of complete and incomplete phrases. Then, participants were asked to listen to the 12 melodies again, but this time, they had to mark the perceived phrase boundaries by pressing a spacebar on a keyboard. Whenever a participant pressed the spacebar, a dot appeared on the screen to signal that the answer was registered. This task was followed by four questions about (1) participants' recognition of the presented melodies, (2) the cues followed to detect phrase boundaries, (3) their liking of the presented melodies, and (4) the difficulty of the task. Questions (1) and (2) were open‐ended. For (3) and (4), participants provided a rating on a 6‐point scale.

### 2.5 Analysis of Behavioral Data

Behavioral data were analyzed in RStudio (R Version 4.1.1, R Core Team, 2021). Responses were time‐logged and assigned to the time window corresponding to the beats in the music to remove excessive fragmentation of the time data.

We focused on (1) the agreement among participants for perceived phrase boundaries, and (2) the accuracy of phrase detections, compared with music theory‐based ground truth.

For (1), we calculated the Krippendorff's alpha (Krippendorff 2011) using RStudio function *kripp.alpha* (*irr* package, RStudio 2021.09.0). The Krippendorff's alpha is a reliability coefficient used to measure the inter‐rater agreement, which ranges from 0 to 1, where 0 means no agreement among participants and 1 describes the absolute agreement.

For (2), we used the *F*‐score, a harmonic mean of precision and recall (Sasaki 2007), where precision is defined as

$$P=\frac{\text{true positives}}{\text{true positives}+\text{false positives}}$$

and recall is defined as

$$R=\frac{\text{true positives}}{\text{true positives}+\text{false negatives}}.$$

The *F*‐score describes how well participants' answers match with the ground truth (phrase boundaries as marked in music score). This measure relies on the positive answers but also takes into account the proportion between correct answers (true positives: recognizing phrase boundaries correctly), incorrect answers (false positives: indicating phrase boundary incorrectly) and phrase boundary omissions (false negatives). Participants were unfamiliar with the musical material presented to them. To account for the potential variability in timing of responses, we implemented a sliding window of three beats around each boundary to capture responses that occurred slightly earlier or later than the boundary itself. The distributions of participants' responses are illustrated for an example stimulus in Supporting Information S1: Figure S4.

In the shuffled condition all phrasal structure was removed by randomizing the notes order in the music material. Consequently, there was no ground truth on which we could base the calculation of the accuracy. In order to compare the regular/irregular and shuffled condition, we applied the ground truth from the original, unshuffled melody to each shuffled melody. Accuracy from the shuffled condition can be interpreted as a baseline accuracy acquired randomly.

Because phrase boundary could be partially predicted with the local acoustic changes (i.e., previous note duration and frequency interval, see Section 2.2), we fitted a model predicting phrase detection rate, using preceding note duration, frequency change, and the interaction between these variables as fixed effects and a stimulus as random effect. Detection rate was calculated as the proportion of participants who identified each boundary.

Additionally, we wanted to explore the relation between the accuracy and musical training of participants. To do so, we correlated the accuracy (*F*‐score) with the results from the Gold‐MSI questionnaire, and more specifically the General Index and Musical Training subscales.

### 2.6 EEG Data Acquisition

The EEG data were recorded using an actiCAP set with 64 active electrodes arranged in the extended 10–20 international system (BrainVision Recorder Professional Version 1.25.0001, Brain Products GmbH). A signal from 62 scalp electrodes was recorded. Additionally, we used one reference electrode (originally PO9), placed at FCz. To provide symmetrical data recording, the signal from electrode PO10 was excluded from the analysis. The impedance levels for the reference electrode were maintained at 20 kΩ as a good level, acceptable between 20 and 50 kΩ. Above 50 kΩ was treated as a bad level. The sampling rate for data acquisition was set to 500 Hz.

### 2.7 EEG Data Preprocessing

Two participants were excluded from the analysis. For one participant, data was not recorded; for the other, the amount of hair did not allow for obtaining a decent EEG signal.

The analysis of the EEG data has been done in MATLAB (MATLAB R2018b), with FieldTrip toolbox (Oostenveld et al. 2011). We used a two‐way band‐pass filter from 0.5 to 35 Hz, order of 5. Epochs were created by matching fragments of the EEG signal with melodies presented in the experiment. Additionally, we extended the epochs by 3 s before the melody onset and 2 s after the offset. The data were re‐referenced to the average activity across all electrodes. To clean the data from the artifacts caused by eye blinks, eye movements, and heartbeat, the independent component analysis (ICA) was performed on the preprocessed EEG data. The average number of rejected components was 3 (SD = 1).

Further, the EEG signal was analyzed in order to extract the activity related to the auditory stimulation and improve the signal‐to‐noise ratio. A common method to improve this is averaging responses across repeated stimuli presentations, but this is not feasible with continuous stimuli like music or speech, spanning over minutes (Crosse et al. 2016) and cannot be practically repeated for typical event‐related potential (ERP) analysis. An alternative approach involves presenting identical stimuli to many participants and averaging their responses, assuming similar brain responses despite individual differences.

To accomplish this, we used multiway canonical correlation analysis (MCCA) (De Cheveigné et al. 2019). The process involved first applying a principal component analysis (PCA) to each participant's EEG data. A second PCA was then performed on these results to extract the shared EEG signal components across participants. This shared component was applied back to the individual data to remove noise. For each participant, we selected the single component that explained the most variance in their data for further analysis. This approach resulted in one EEG signal per participant, rather than multi‐channel data that would be available without this denoising step. Further analyses focused on the first MCCA component, which explained the largest proportion of variance and exhibited an auditory topographical distribution (see Supporting Information S1: Section S5 and Figure S6).

### 2.8 Temporal Response Function (TRF) Over Acoustic Envelope

The TRF was first used to observe how the envelope of presented musical material is modulating the neural signal obtained with EEG (Crosse et al. 2016). The ERP method has a long tradition as a way of describing a neural response to certain features of the stimulus presented throughout the experiment (Sur and Sinha 2009). However, its usability is quite limited in the context of studies based on continuous material, such as natural speech (Brodbeck et al. 2018; Teng et al. 2021) or music (Di Liberto et al. 2020; Leahy et al. 2021), where now the TRF is used more widely.

The TRF models the linear mapping between a stimulus feature and the evoked neural response. Conceptually, the forward TRF model describes how the brain transforms acoustic input into neural activity (Crosse et al. 2016). Under this assumption, a salient stimulus feature, such as the acoustic envelope, is represented in the EEG in a linear and temporally stable manner across the experiment. Estimating the TRF therefore allows finding the set of temporal weights that best predict the EEG signal from the stimulus feature(s).

For TRF analysis, the acoustic envelope was extracted for each stimulus using a gammatone filterbank (32 bands) spanning 50–4000 Hz to model cochlear frequency selectivity. Each stimulus was sampled at 16 kHz. The resulting envelope was downsampled to 100 Hz to match the sampling rate of the EEG signal and applied as a single predictor for the TRF analysis. The first and last seconds of the neural data were removed to avoid potential influence of the stimulus onset or offset. TRF analysis was performed in the window between 200 ms before and 500 ms after acoustic stimulation, but for statistical analysis the window was narrowed to −100 to 400 ms. The regularization of TRF was applied as described in Supporting Information S1: Section S7.

### 2.9 Cerebral–Acoustic Coherence (Cacoh)

To quantify the synchronization between the neural signal and the acoustic envelope, we computed Cacoh, a measure of coupling between EEG activity and amplitude fluctuations in the stimulus in the spectral domain (Doelling et al. 2014; Teng et al. 2024). This measure reflects the extent to which neural activity entrains to temporal modulations in the sound, providing an index of the strength of neural tracking of the envelope. Cacoh increases with attention to the auditory stimulus (Sturm et al. 2015). Also, it can be generally interpreted as reflecting the reliability of neural encoding of acoustic temporal structure.

Cacoh was calculated separately for each stimulus to avoid biases that might arise from analyzing melodies of different lengths within the entire musical material (Teng et al. 2024). We used magnitude‐squared coherence, which measures the similarities in frequency content between two signals (Malekpour et al. 2018), implemented via the *mscohere* function in MATLAB. Both neural and acoustic signals (temporal envelope of stimuli, representing amplitude modulation over time) were downsampled to 100 Hz, and the first and last second of each signal were removed to reduce the effects of stimulus onset and offset. The analyzed frequency range was set between 0.1 and 20 Hz with 0.05 Hz steps. Coherence was calculated using a Hanning window of 1024 points with 512‐point overlap. Cacoh values were averaged across participants for each condition, and differences between conditions were assessed using pairwise *t*‐tests with Bonferroni correction.

### 2.10 Neural Tracking of Phrase Structure

In a recently published paper, Teng et al. (2024) describe neural signals locking to the phrase boundaries. Following their methods, we measured the effect of phrasal structure on the neural responses at the beat rate. Although the intended beat rate was 74.4 bpm, which would correspond to the frequency of 1.24 Hz, we do not observe strong tracking of this frequency (see Section 3 for Cacoh). Due to the high granularity of the music notes, that is, notes much shorter than the beat rate in the musical material, the beat frequency was not represented strongly enough (see Figure 1C for the spectral analysis of the acoustic envelope of the stimuli). Hence, we consider a double‐beat rate (2.48 Hz), corresponding to the duration of a quaver, as frequency used in the main analysis. Following the method proposed in the original paper, we observed the temporal dynamics in the EEG power modulation at the double‐beat rate.

To measure EEG power modulation around phrase boundaries, we conducted a TRF analysis of the EEG power envelope at the double‐beat rate, using phrase boundary as a predictor. Additionally, we included the note duration and frequency as predictors in the TRF model, in order to control for a possible effect of local changes on the brain response to the phrase boundary. The TRF was calculated on the EEG power envelope at 2.48 Hz, for each condition, for the time window of 6 s, from 3 s before to 3 s after a phrase boundary, to observe the temporal dynamics of the effect. EEG power envelopes were extracted using time‐frequency analysis implemented in *FieldTrip* (Oostenveld et al. 2011). We applied Morlet wavelet decomposition using *ft_freqanalysis* to compute power spectral estimates at frequencies of interest (1, 1.24, 2, 2.48, and 3–35 Hz). To balance temporal and spectral resolution across frequencies, we used frequency‐dependent wavelet widths, ranging from 3 cycles for low frequencies (1–2.48 Hz) and increasing widths up to 10 cycles for higher frequencies. This approach provides better temporal resolution at lower frequencies relevant for phrase tracking. Power values were baseline‐corrected by computing the mean power in a pre‐stimulus baseline period. For the phrase boundary TRF analysis, we extracted the power envelope at 2.48 Hz for each trial and subject. The resulting time series had a sampling rate of 100 Hz, matching the temporal resolution of the stimulus predictors.

We also introduced two control conditions to assess the reliability of our measures. The shuffled condition consisted of fragmented and randomly reordered versions of the original stimuli. Because these shuffled sequences no longer contained meaningful phrase boundaries, we generated a corresponding sequence of arbitrary phrase markers by taking the boundary timings from the original stimuli used to create the shuffled melody. In addition, we computed a TRF for the regular and irregular conditions using randomly selected onsets as phrase boundaries. Together, these control analyses ensured that (a) the temporal placement of boundaries alone did not drive the observed effects, and (b) the measured responses were not merely evoked by any note onset in the stimuli. Additionally, we used the model with randomly assigned phrase boundaries to construct a null distribution. This was to create a threshold to which we could compare the performance of the models including actual phrases as predictors. To do so, we repeated the model estimation 100 times, each time permuting the boundary onsets. Model performance across these permutations defined a null distribution, from which we derived a significance threshold by taking the 95th percentile. Model performance that exceeded this threshold was interpreted as indicating the meaningful addition of phrase boundary information as a predictor.

To compare the strength of a modulation between conditions, we calculated RMS of the TRF weights for each condition in the two‐beat window between beat − 1 and beat + 1. RMS for conditions was compared using pairwise *t*‐test comparison, Bonferroni corrected. We further explored the temporal dynamics of EEG power modulation by examining peak latency across conditions. For each participant, we calculated the time of the peak modulation and compared these values between conditions using a *t*‐test to measure the difference.

We also included a control analysis to ensure that the observed changes in EEG power could be attributed specifically to the frequency of double‐beat rate. We computed a TRF using the 1 Hz EEG power envelope, again using phrase boundaries as the predictor. This provided a comparison at a frequency close to the beat or double‐beat rate, but not musically meaningful for the stimuli.

All the *R* ^2^ values for the TRF models have been computed as a proportion of variance of predicted EEG signal explained by the actual EEG signal, using *mTRFpredict* function from the *mTRF* toolbox (Crosse et al. 2016) in MATLAB.

## 3 Results

### 3.1 Listeners Are Able to Detect Phrase Boundaries

**FIGURE 2:**
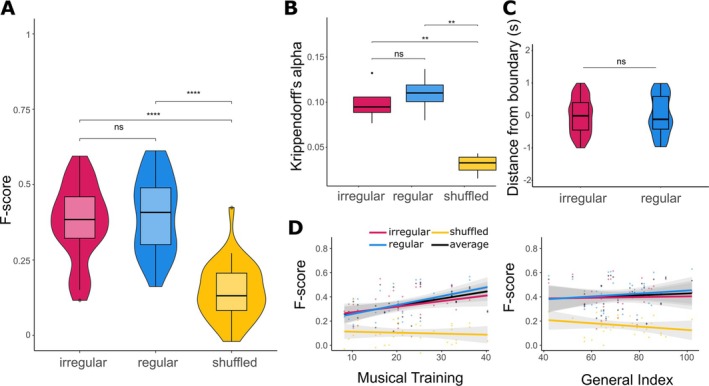
Results of the behavioral task. (**A**) Accuracy of participants' phrase boundary detection, represented by the *F*‐score. There was no significant difference between regular and irregular conditions while they both showed higher *F*‐score than in the shuffled condition (i.e., the baseline accuracy). (**B**) Agreement between participants, measured with Krippendorff's alpha (values ranging between 0 and 1). There was no significant difference between regular and irregular conditions but agreement in both conditions was significantly higher than agreement in the shuffled condition. (**C**) The timing of correctly detecting a phrase boundary between conditions. (**D**) Relation between participants' accuracy scores and their musical training as well as the general index of musical sophistication, as estimated with the Gold‐MSI questionnaire. A correlation was found between accuracy scores and Musical Training subscale. There is no correlation between General Index of musicality and accuracy results.

Participants signalled the detection of phrase boundaries by pressing a button in three conditions: (1) regular, (2) irregular, and (3) shuffled (control conditions, where notes of regular and irregular conditions were randomly shuffled to disrupt musical structures). Listeners ability to explicitly identify phrases was affected by the condition (repeated‐measure ANOVA on accuracy level across conditions, *F*_(2,66)_ = 91.317, *p* < 0.0001, $\eta_{g}^{2}$ = 0.499, Figure 2A). Post hoc pairwise *t*‐test comparison (Bonferroni‐corrected) revealed that there was no significant difference between regular and irregular condition (*p*_*adj*_ = 0.444), whereas there was a difference between these and the shuffled condition (irregular—shuffled: *p*_*adj*_ < 0.0001; regular—shuffled: *p*_*adj*_ < 0.0001). Various lengths of phrases in stimuli (3 to 30 beats) did not lead to increased difficulty than when the phrases are always eight beats long.

This finding was confirmed with the Krippendorff's alpha, which quantified the agreement between participants regarding the placement of phrase boundaries. The general agreement across all conditions was rather low (M = 0.08, SD = 0.04, for the measure ranging between 0 and 1). The ANOVA comparing the agreement across conditions revealed differences (*F*)_(2,9)_ = 17.59, *p* < 0.001, $\eta_{g}^{2}$ = 0.8, Figure 2B. As for the accuracy, the post hoc Tukey HSD comparison confirmed that although there was no significant difference in agreement between the regular and irregular conditions (*p*_*adj*_ = 0.782), both the regular and irregular conditions had significantly higher agreement than the shuffled condition (irregular—shuffled: *p*_*adj*_ = 0.003; regular—shuffled: *p*_*adj*_ = 0.001).

Participants' responses closely matched the timing of the actual phrase boundaries (Figure 2C). One‐sample *t*‐tests against zero revealed no significant deviation from zero in either condition (regular condition: *t*_(29)_ = −0.28, *p* = 0.78; irregular condition: *t*_(34)_ = −0.38, *p* = 0.71). The difference in degrees of freedom reflects five participants who did not produce any correct detections of phrase boundary in the regular condition. The temporal dynamics of correctly detected boundaries were comparable across conditions: on average, participants responded 18 ms before the boundary in the irregular condition and 21 ms before the boundary in the regular condition. A paired *t*‐test comparing these offsets showed no significant difference between conditions (*t*_(29)_ = −0.295, *p* = 0.77).

Though the pattern of results is clear at the group level, strategies in answering differed strongly between participants (Supporting Information S1: Figure S4), with some participants responding very frequently throughout the task and others rather sparsely. We thus explored further the relation between participants' accuracy and their musicality as well as musical training. Although the general index from the Goldsmith Music Sophistication Index (Müllensiefen et al. 2014) did not correlate with the phrase perception ability (*r*_(33)_ = 0.475, *p* = 0.638), we observed a correlation between the accuracy and the Musical Training subscale (*r*_(33)_ = 0.456, *p* < 0.001, Figure 2D). See Supporting Information S1: Section S8 for detailed results for each condition.

### 3.2 Phrase Boundary Detection Relies on Structural Cues Rather Than Local Acoustic Changes

The analysis of local acoustic cues (longer note durations and frequency interval between notes) revealed that occurrence of a boundary can be predicted with those local cues (see Section 2). To test whether participants were relying on local acoustic cues or rather on higher level structure when identifying the phrases, we fitted a linear model predicting boundary detection accuracy (a proportion of participants who correctly identified phrase boundary) from the acoustic features. The model revealed no significant contribution of these local features to detection accuracy. Neither the frequency interval (*β* = 0.02, 95% CI [−0.01, 0.09], *p* = 0.17), nor its interaction with note duration (*β* = 0.001, 95% CI [−0.01, 0.01], *p* = 0.91) significantly predicted performance. Previous note duration showed a marginal effect (*β* = 0.02, 95% CI [0.00, 0.05], *p* = 0.048). The overall model explained only 6.7% of variance in detection accuracy (adjusted *R* ^2^ = 0.046) and marginally reached statistical significance (*F*_(3,134)_ = 3.214, *p* = 0.025). These findings suggest that although phrase boundaries were associated with local acoustic features, participants did not fully rely on these features when detecting boundaries.

### 3.3 Neural Tracking of Phrase Boundaries Is the Strongest for Regular Phrases

The first step of the EEG data analysis was to compare the neural tracking of beat for the proposed music material across conditions, in order to assess whether coherence between the brain activity and acoustic envelope was affected by the temporal regularity of the material. We did not expect any differences between irregular and regular conditions because both conditions were derived from very similar original material. This analysis was conducted to rule out that possible differences in phrase boundary tracking could be explained by weaker neural coupling at the beat or double‐beat frequency.

**FIGURE 3:**
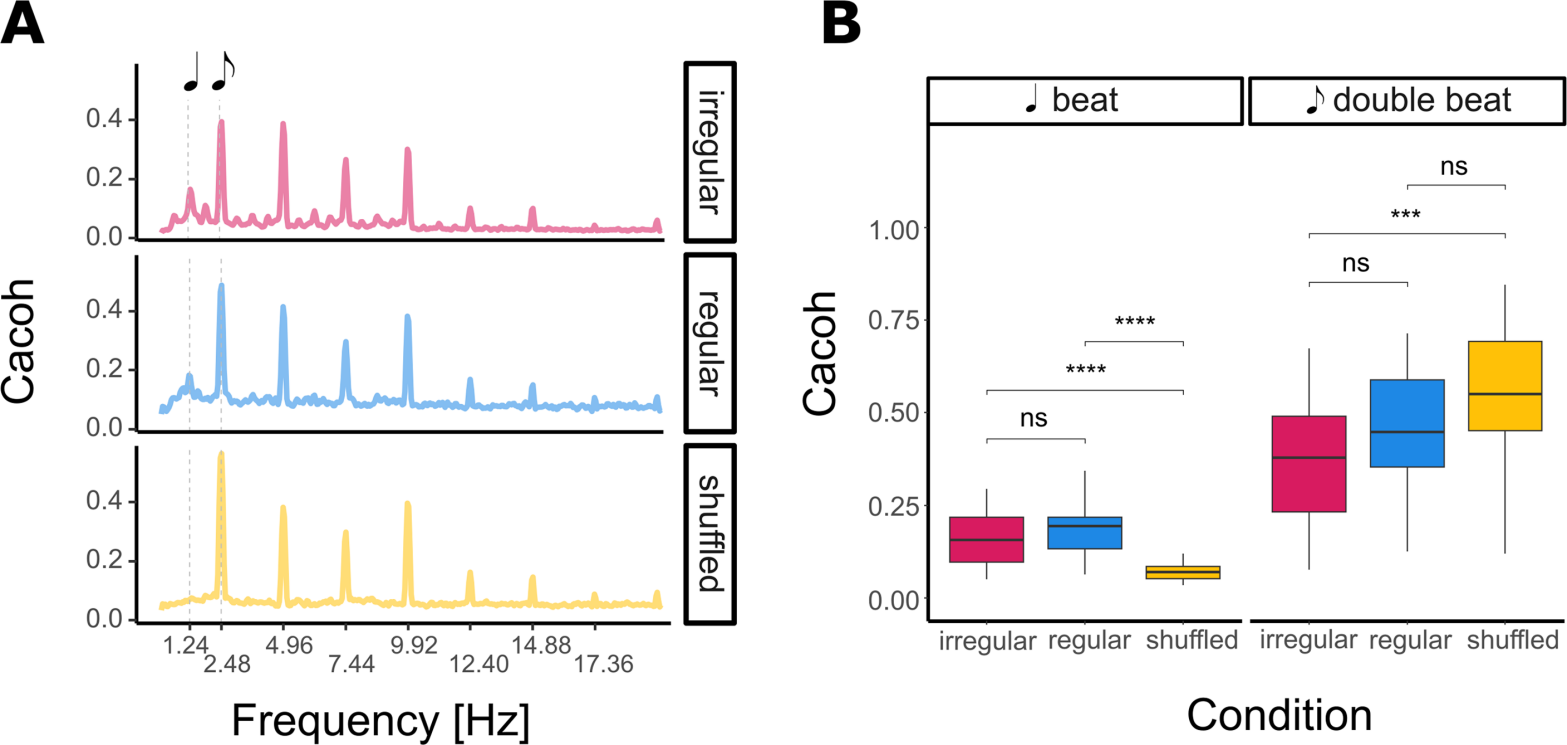
Results of the time‐frequency coupling between stimuli and the EEG data. (**A**) Cerebral–acoustic coherence (Cacoh) at beat rate (1.24 Hz) and its harmonics. (**B**) Comparison between Cacoh values across conditions at beat (1.24 Hz) and double‐beat rate (2.48 Hz).

We computed Cacoh (Doelling et al. 2014) to quantify the coupling between frequencies in the neural and acoustic signal (Figure 3A). A frequency corresponding to the beat rate in the stimuli was 1.24 Hz, and 2.48 Hz would be the double‐beat rate (the frequency corresponding to the quaver notes in the stimuli). We measured the beat tracking and compared the Cacoh values for the beat and double‐beat rates to observe the effect of condition on the beat and double‐beat tracking. Pairwise *t*‐test comparison shows no significant difference between regular and irregular conditions; however, for the shuffled condition, beat tracking was significantly lower, and double‐beat tracking significantly higher (Figure 3B and detailed results in Supporting Information S1: Section S10). This likely resulted from the method used to create the shuffled stimuli: By dividing the original melodies into equally long units, we increased the occurrence of double‐beat notes while removing those with longer durations (see Figure 1C).

Overall, the neural tracking of beat rate in both regular and irregular conditions showed no differences, confirming that in both conditions coupling between frequencies present in the acoustics material and brain activity was similar. Removing the structure of music completely in shuffled condition altered how these lower‐level features of sounds are processed, which likely arises from the differences in the distribution of note durations between regular/irregular and shuffled conditions.

In addition, following the original analysis pipeline of Teng et al. (2024), we analyzed broadband neural tracking of acoustic envelope to observe potential differences in envelope tracking. This analysis revealed comparable envelope tracking between regular and irregular conditions, with slightly weaker responses for the shuffled condition (Supporting Information S1: Section S9).

**FIGURE 4:**
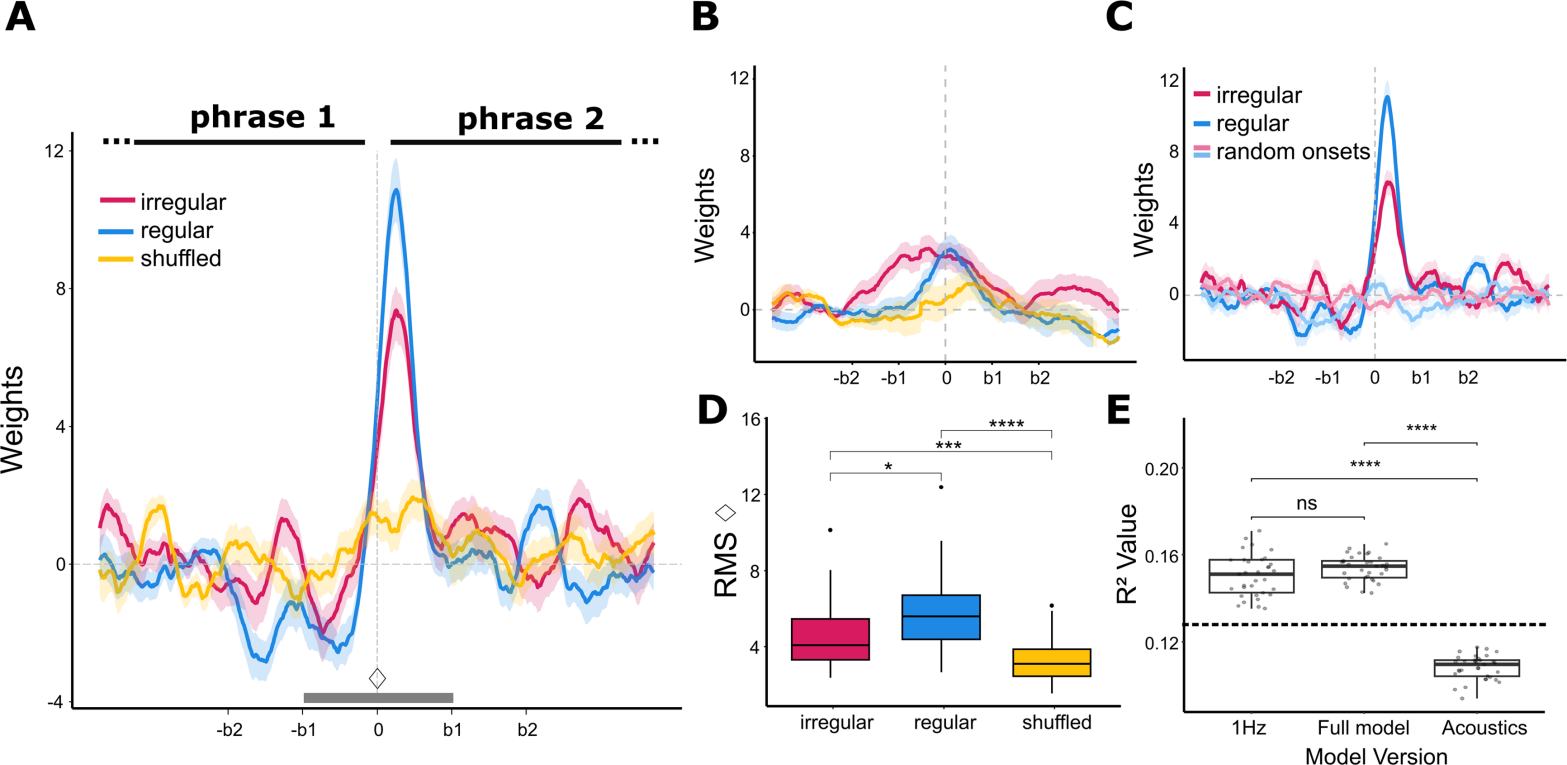
Results of the TRF analysis over phrase boundary. (**A**) TRF computed on the EEG power envelope at the double‐beat rate (2.48 Hz). The TRF captures the modulation of EEG power from 3 s before to 3 s after the phrase boundary (0 s). Beat positions relative to the boundary are shown on the x‐axis (b). (**B**) TRF weights for the three conditions computed on the EEG power at 1 Hz. Here, the phrase boundary predictor was applied to the EEG power envelope at 1 Hz, producing comparatively flatter and weaker responses. (**C**) Control analysis using random onsets. TRF weights obtained using the true phrase boundaries (2.48 Hz) are contrasted with weights obtained when random onsets were used as the phrase‐boundary predictor. (**D**) RMS across conditions was computed to represent the strength of the TRF weights around the phrase boundary (from ‐b1 to b1, marked with a grey band and the 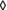 shape), showing differences between all conditions. (**E**) Model comparison. Prediction performance (*R* ^2^) between the predicted and observed EEG signals for four TRF models: a 1 Hz model (EEG power envelope at 1 Hz; predictors: note duration, note frequency, and phrase boundaries), a full model (EEG power envelope at 2.48 Hz; predictors: note duration, note frequency, and phrase boundaries), and an acoustics‐only model (EEG power envelope at 2.48 Hz; predictors: note duration, note frequency). The dashed line marks the threshold value obtained by taking the 95th percentile of *R* ^2^ values computed with 100 permutations for the model with randomized boundary predictors.

Most importantly, we were interested in the neural tracking of higher‐level structures in music—phrases. To quantify that we used EEG power modulation at the double‐beat rate, following the procedure of Teng et al. (2024). We performed a TRF analysis on the EEG power envelope at the double‐beat rate (2.48 Hz), using phrase boundaries as a regressor, obtaining the modulation of the power around the phrase boundary (Figure 4A). We also added note frequency and note duration as regressors, to account for the local changes in the stimuli. We obtained predicted brain response around the phrase boundary. Using RMS of the TRF weights around the phrase boundary we compared the tracking of phrasal structure across conditions. Repeated‐measures ANOVA results confirmed the main effect of condition (*F*_(2,64)_ = 26.02, *p* < 0.0001, $\eta_{g}^{2}$ = 0.258). Pairwise *t*‐test comparison revealed significant differences between all conditions (irregular—regular: *p*_*adj*_ = 0.019; irregular—shuffled: *p*_*adj*_ = 0.001; regular—shuffled: *p*_*adj*_ < 0.0001; Figure 4D). To test whether each condition exhibited significant neural responses to phrase boundaries, we performed cluster‐based permutation tests against zero on the TRF weights with 1000 permutations. The regular condition showed three significant clusters. The primary cluster extended from −11 to 54 ms (*p* = 0.001), capturing the immediate response around the phrase boundary. Two additional smaller clusters emerged: one preceding the boundary (−68 to −23 ms, *p* = 0.024) and one following it (158–195 ms, *p* = 0.045). The irregular condition revealed one significant cluster from −8 to 57 ms (*p* = 0.001), closely matching the timing of the primary cluster in the regular condition. The shuffled condition showed no significant clusters, confirming that TRF weights remained around zero when phrasal structure was removed.

Additionally, we analyzed the difference in modulation latency between the regular and irregular conditions. A paired *t*‐test confirmed that peak modulation latency did not differ between conditions (*t*_(32)_ = −0.42, *p* = 0.667).

To evaluate the specificity of these effects, we performed several control analyses. First, we repeated the TRF analysis using EEG power envelopes at 1 Hz, a frequency with no musical relevance, with phrase boundaries, note frequency and note duration as predictors. As expected, the resulting TRF weights showed minimal modulation around phrase boundaries and were substantially flatter than in the main analysis (Figure 4A,B). However, the predictive power of the model (*R* ^2^ = 0.150 ± 0.078) was similar to the full model computed at the 2.48 Hz frequency, which explained about 15% (*R* ^2^ = 0.154 ± 0.072; Figure 4E). For comparison, model including only note frequency and note duration explained roughly 11% of variance (*R* ^2^ = 0.108 ± 0.056). RMS values around the phrase boundary for weights at 1 Hz showed no differences between conditions (*F*_(2,64)_ = 0.452, *p* = 0.64, $\eta_{g}^{2}$ = 0.01). Taken together, we can conclude that, although some of the phrase‐related activity might occur at 1 Hz, the strong effect is observed when using the double beat rate as the frequency.

For the final control analysis, we computed TRFs using random onsets as boundary predictor. The model also included note frequency and note duration as predictors. The model performance was significantly worse, as compared with the full model (*R* ^2^ *=* 0.128 ± 0.06; *t*_(33)_ *=* 30.5, *p*_*adj*_ < 0.0001). No interpretable structure or modulation was observed (Figure 4C), which confirms that the main effects observed can be attributed to the phrase rather than to any note onset.

### 3.4 Participants Without Musical Expertise Can Describe Segmentation Cues

After completing the entire experiment, non‐musical participants (see Section 2) were asked to name the cues they used while performing the behavioral part of the task—detecting musical phrases. Although lacking formal musical education, some individuals were able to accurately describe phrase segmentation cues. Nine out of 35 participants answered that they did not recognize any specific cues or that they were just following their feeling; but others often mentioned the presence of a pause at the end of a phrase (which we would interpret as a long note at the end of a phrase because a silent break within a stimulus was not introduced when presenting the stimuli). Participants also noticed latent cadenzas, or closing harmony, changes in the melody or phrase as a closed unit. Only one participant mentioned a cue related to the regularity of the stimulus, namely, that the phrase started with the upbeat, suggesting that the phrase boundary should start at the beginning of the measure. Notably, no participants mentioned the regularity of phrase length as a cue, indicating that phrase boundaries were not anticipated based on regular time intervals and supporting the empirical findings reported from both the behavioral and neural tracking results.

## 4 Discussion

Literature on phrase perception showed that both lay and expert listeners are able to identify music phrases based on local (acoustic) and global (harmonic) cues (Burunat et al. 2024; Knösche et al. 2005; Neuhaus et al. 2006; Tan et al. 1981). However, due to the very regular temporal structure of music traditionally used in such experiments, to which listeners are highly sensitive (Andreou et al. 2011; Large and Palmer 2002), it is difficult to disentangle the contribution of temporal regularity to the segmentation of music. By proposing music pieces containing phrases of different lengths, our study explores the effect of temporal regularity on the segmentation of continuous streams of music. Results showed that listeners are capable of identifying musical phrases irrespective of their temporal regularity and that the additional cue of temporal regularity does not further improve accuracy. This indicates that the recognition of phrase boundaries and the segmentation of music are driven not only by the temporal predictability but also by the understanding of musical cues.

Consistent with the accuracy results, where participant responses were compared with phrase boundaries defined by music theory, the inter‐individual agreement analysis shows that removing temporal regularity does not make phrase cues harder to interpret. Inter‐individual agreement was significantly and equally higher in the two conditions with musical structure (regular and irregular) compared with the shuffled condition, where local acoustic cues were randomized and the harmonic structure was disrupted. This implies that participants responded randomly, without interpreting acoustic changes as musical cues or imposing regular time intervals when segmenting material which did not contain a specific structure. Note however that the overall agreement, which captures the extent to which participants share a common perception of musical structure, was modest (significant Krippendorff's alpha is usually between 0.4 and 0.75; Koops et al. 2019), likely due to individual differences among participants in their strategies to perform the task, that is, reporting either several or seldom phrase boundaries.

Our results support that participants' detection of phrase boundaries relies on inferred harmony and perceived melodic completeness (Nattiez 1990; Tan et al. 1981), rather than pronounced local acoustic features or solely temporal predictions. Because our participants were lay listeners, these results show that one can develop an understanding of musical structure in accordance with musical theory through mere exposure to music, which is in line with previous literature (Burunat et al. 2024; Deliège 1987; Hansen et al. 2021; Quiroga‐Martinez et al. 2020; Sridharan et al. 2007; Teng et al. 2024; Tillmann et al. 1998). We also confirm that the recognition of phrase boundaries did not solely rely on local changes (namely, longer note durations and interval jumps). Neither note duration nor note frequency strongly predicted participants' phrase‐recognition accuracy, and the model, including these cues, was only marginally significant. Yet, in the stimuli themselves, phrase boundaries were highly predictable from these acoustic features. This indicates that, although local acoustic changes may have contributed slightly to participants' decisions, they were not the primary cues guiding phrase segmentation. Instead, participants appeared to rely on their understanding of the musical sequence, its contour (Dowling and Fujitani 1971), and harmonic progression (Koelsch 2011; Lerdahl and Jackendoff 1983; Teng et al. 2024), rather than responding only to the local changes in the melody.

We observed a correlation between musical training (defined as results of a Musical Training subscale within Gold‐MSI questionnaire) and accuracy, particularly in the case of the regular condition. This may be attributed to the general tendency of individuals with musical expertise to demonstrate heightened sensitivity to pattern detection (Schön and François 2011), better perception of time intervals (Thibault et al. 2024), or detection of temporal regularities (Van Zuijen et al. 2005). Overall, musical training was associated with higher accuracy in detecting phrase boundaries. This aligns with previous findings showing that, although non‐musicians can perform the task, trained musicians achieve noticeably higher accuracy (Neuhaus et al. 2006; Zhang et al. 2016).

The timing of both the behaviorally reported phrase boundaries and the peak TRF weights indicates that participants detected phrase boundaries at the moment they occurred, without showing strong anticipatory tendencies, as it was observed in some previous studies (Burunat et al. 2024; Hansen et al. 2021; Teng et al. 2024). This is likely due to the higher complexity of our musical material, which provided fewer clear cues that would allow listeners to predict phrase endings well in advance.

Importantly, EEG power modulation confirmed the behavioral results, with a pronounced increase in activity around the boundary between two phrases in both regular and irregular conditions, while we did not observe spontaneous activity at regular times in the shuffled condition (where studies in speech suggest that when clear cues are absent, listeners rely on temporal regularity) (Meyer et al. 2017). Because participants were not told to focus on music structure and were not given any explicit definition of a musical phrase, the result suggests the implicit and uninformed perception of musical structure. The timing of the modulation peak did not differ across conditions but we observed a difference in modulation strength between regular and irregular phrases. This difference is not reflected in the behavior (i.e., the accuracy of phrase detection does not differ between these conditions) and could indicate that there might be a threshold of activity at which the explicit decision to place the boundary is made. Therefore, temporal regularity might provide an additional cue that aids in segmentation, though not strongly enough to be reflected in behavioral accuracy or agreement measures.

## 5 Conclusions

This study confirms the remarkable human ability to detect patterns in complex acoustic stimuli without explicit knowledge of underlying rules and demonstrates that this ability persists even when temporal regularity is disrupted. Although regularity seems to increase the strength of neural tracking of phrases, our results support that it is not a determining factor for the segmentation processes. Instead, we provide evidence that listeners use their encultured understanding of theoretical rules relative to musical structure to guide segmentation, a mechanism that fundamentally contributes to music processing and appreciation.

## Author Contributions

**Zofia Anna Hołubowska:** conceptualization, data curation, formal analysis, investigation, methodology, visualization, writing – original draft, writing – review and editing. **Xiangbin Teng:** methodology, software, writing – review and editing. **Pauline Larrouy‐Maestri:** conceptualization, funding acquisition, methodology, project administration, resources, writing – original draft, writing – review and editing.

## Funding

X.T. was supported by the Improvement on Competitiveness in Hiring New Faculties Funding Scheme, the Chinese University of Hong Kong (4937113).

## Conflicts of Interest

The authors declare no conflicts of interest.

## Supporting information


**Data S1:** Supporting information.

## Data Availability

Behavioral data, as well as preprocessed EEG data, stimuli, and preprocessing and analysis scripts are available at https://osf.io/btmxa. Raw EEG data is available upon request to the corresponding author (Z.A.H.).
