# Supplementary material for: Neural and Behavioral Tracking of Musical Phrases Occurs Without Temporal Regularity
Source: Eur J Neurosci. 2026 Apr 12;63(7):e70481. doi: 10.1111/ejn.70481 (PMC13071237; doi:10.1111/ejn.70481)
Supplement: Supplementary file 1 — Data S1: Supporting information. [file EJN-63-0-s001.docx]

Supplementary materials for

**Neural and behavioural tracking of musical phrases occurs without temporal regularity**

Zofia Anna Hołubowska^1,2,3,4^, Xiangbin Teng^5,6^, Pauline Larrouy-Maestri^1^

^1^ Max-Planck-Institute for Empirical Aesthetics, Frankfurt am Main, Germany

^2^ Institute of Applied Psychology, Faculty of Management and Social Communication, Jagiellonian University, Kraków, Poland

^3^ Max-Planck-Institute for Human Cognitive and Brain Sciences, Leipzig, Germany

^4^ Institute of Biology, Faculty of Life Sciences, University of Leipzig, Leipzig, Germany

^5^ Department of Psychology, The Chinese University of Hong Kong, Shatin, N.T., Hong Kong SAR, China

^6^ Brain and Mind Institute, The Chinese University of Hong Kong, Shatin, N.T., Hong Kong SAR, China

Corresponding author:

Zofia Anna Hołubowska

Institute of Biology, Faculty of Life Sciences, University of Leipzig, Leipzig, Germany

Talstraße 33

04103 Leipzig

Germany

holubowska@cbs.mpg.de

**S1.** A list of all pieces by Johann Sebastian Bach used to create the stimuli:

Das Wohltemperierte Klavier BWV 846–893

8 Kleine Präludien und Fugen, BWV 553-560

Fantasia and Fugue in G minor, BWV 542

Fugue in A minor, BWV 947

Fugue on a Theme by Giovanni Legrenzi, BWV 574

Fugue on a Theme by Corelli, BWV 579

Fugue in G minor, BWV 578

Fugue in G major, BWV 576

Prelude and Fugue in E minor, BWV 533

Prelude and Fugue in C major, BWV 547

Prelude and Fugue in C Major, BWV 545

Prelude and Fugue in C Major, BWV 531

Inventions and Sinfonias, BWV 772-801

Prelude and Fugue in G minor, BWV 535

Die Kunst der Fuge, BWV 1080

Prelude and Fugue on 'B-A-C-H', BWV 898

Prelude and Fughetta in F major, BWV 901

Fantasia and Fugue in A Minor, BWV 904

Fantasia and Fugue in C minor, BWV 906

Fantasia and Fughetta in B-flat Major BMW 907

Concerto and Fugue in C Minor, BWV 909

Fugue in E Minor, BWV 945

Fugue in C Major, BWV 946

Fugue in A Major, BWV 949

Fugue in A Major, BWV 950

Fugue in B Minor, BWV 951

Fugue in C Major, BWV 952

Sonatas and partitas for solo violin, BWV 1001–1006

Concerto for 2 Harpsichords in C Major, BWV 106

**S2.** Phrasal structure of the melodies used as stimuli in the experiment.

| Stimulus | Length of consecutive phrases |
| --- | --- |
| regular_maj_1 | 8 8 8 8 8 8 8 8 12 8 |
| regular_maj_2 | 8 8 8 8 8 8 8 8 8 |
| regular_min_1 | 8 8 8 8 8 8 8 8 8 8 8 8 8 |
| regular_min_2 | 8 8 8 8 8 8 8 8 8 8 8 8 |
| irregular_maj_1 | 5 7 7 10 7 8 12 18 5 7 8 12 25 7 8 8 7 12 8 7 7 16 13 |
| irregular_maj_2 | 6 18 12 8 12 5 7 6 10 9 7 8 9 7 24 8 4 7 7 10 30 10 7 8 |
| irregular_min_1 | 12 8 13 15 12 12 5 11 16 7 8 16 16 7 17 3 4 8 9 11 20 12 8 12 16 |
| irregular_min_2 | 6 6 9 10 10 9 13 16 6 8 10 16 4 10 14 12 7 12 16 14 18 16 6 10 |

**S3. Creation of the stimuli**

When creating the irregular stimuli, we considered the placement of a phrase beginning in the original piece of music (i.e., at which beat in the bar the phrase starts). In music, there is a sequence of beats, which could be described as strong or weak. In the metre of $\frac{4}{4}$, which was used in our musical material, a bar consists of four beats - the first beat is the strongest, followed by a weak one, then stronger, and again weaker (**1** - 2 - **3** - 4). Note that the strength of a beat is perceived by listeners without increased loudness of a sound^1^, which supports that keeping the original placement of the phrase beginning within a bar is relevant here.

**S4.** **Example behavioural data from participants** (N = 35). Upper panel represents data summed for one of the stimuli – the y axis shows the number of participants who reported detecting a phrase boundary. Responses from individual participants are reported in the lower panel. Vertical lines mark phrase boundaries present in the musical material.


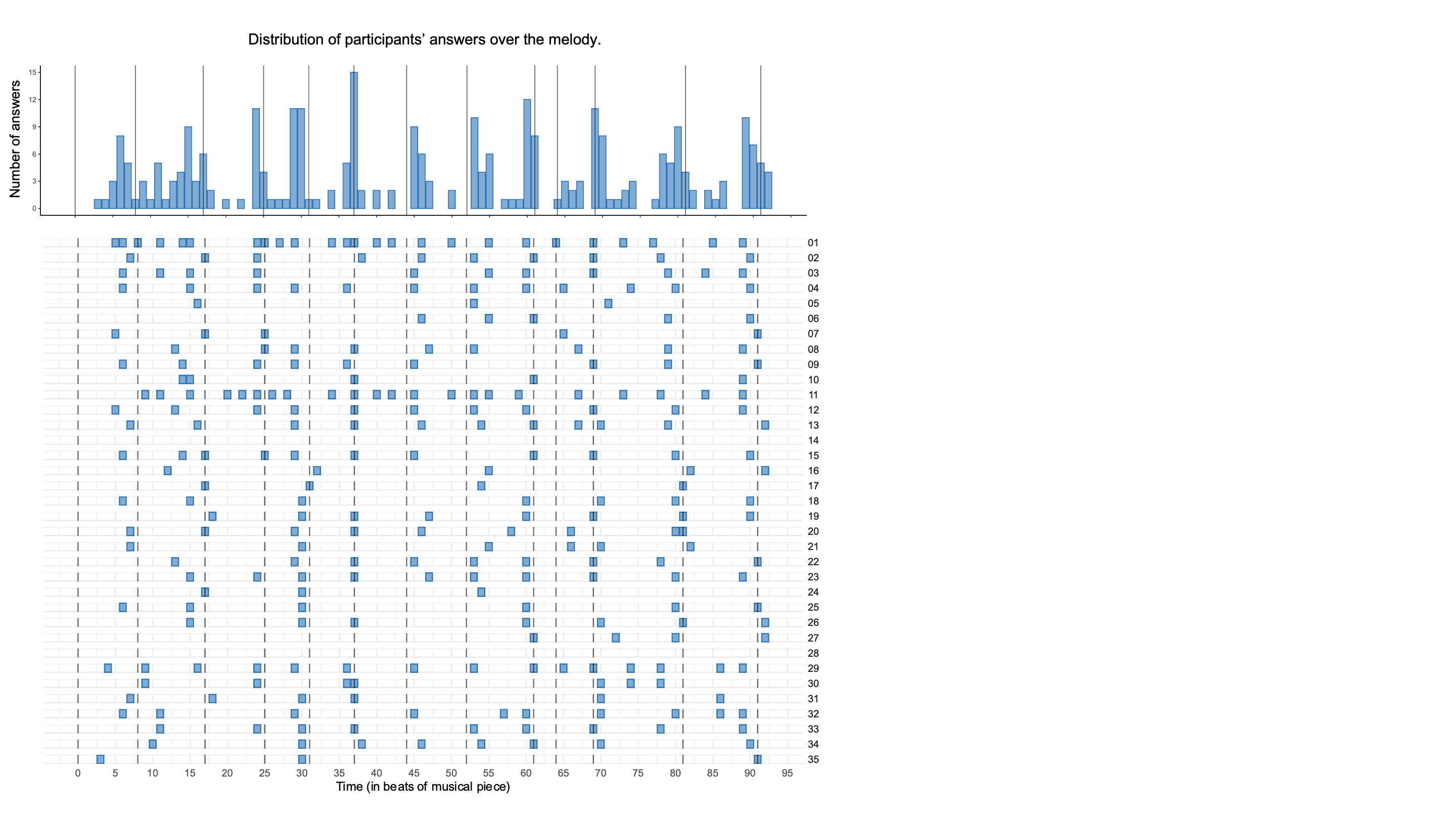


**S5. Preprocessing of EEG signal – Multivariate Canonical Correlation Analysis (MCCA)**

Following the concerns regarding preprocessing and averaging EEG data in the context of continuous acoustic signal, such as speech and music^2,3^, we propose to use a canonical correlation analysis (CCA), which involves the identification of linear associations between two multidimensional matrices^4^. This approach is only suitable for a pair of subjects, so multiway canonical correlation analysis (MCCA) is suggested for working with multi-subject datasets. First, we applied principal component analysis (PCA) to EEG recording of each participant. Then, we perform a second PCA on the results from the previous step, in order to obtain the shared component of the signal from all participants. The results of the second PCA are applied back to the EEG data from single participants, in order to remove the noise. A more detailed description and application of MCCA can be found in De Cheveigné *et al*.^2^

To implement MCCA, epochs were first extracted from a continuous recording for each participant. Each epoch included 3 seconds before and after the stimulus, where one stimulus is one melody (approx. 2 minutes long). MCCA was then applied to obtain 50 components, from which the one that explained the most variance was selected. This selected component was then applied to each participant's data. The signal was further segmented into smaller fragments, each representing different melodies used in the study.

**S6. Variance profile of MCCA components**. Spectral and topographical analysis of the first three MCCA components. The first one was used in the analysis.

**
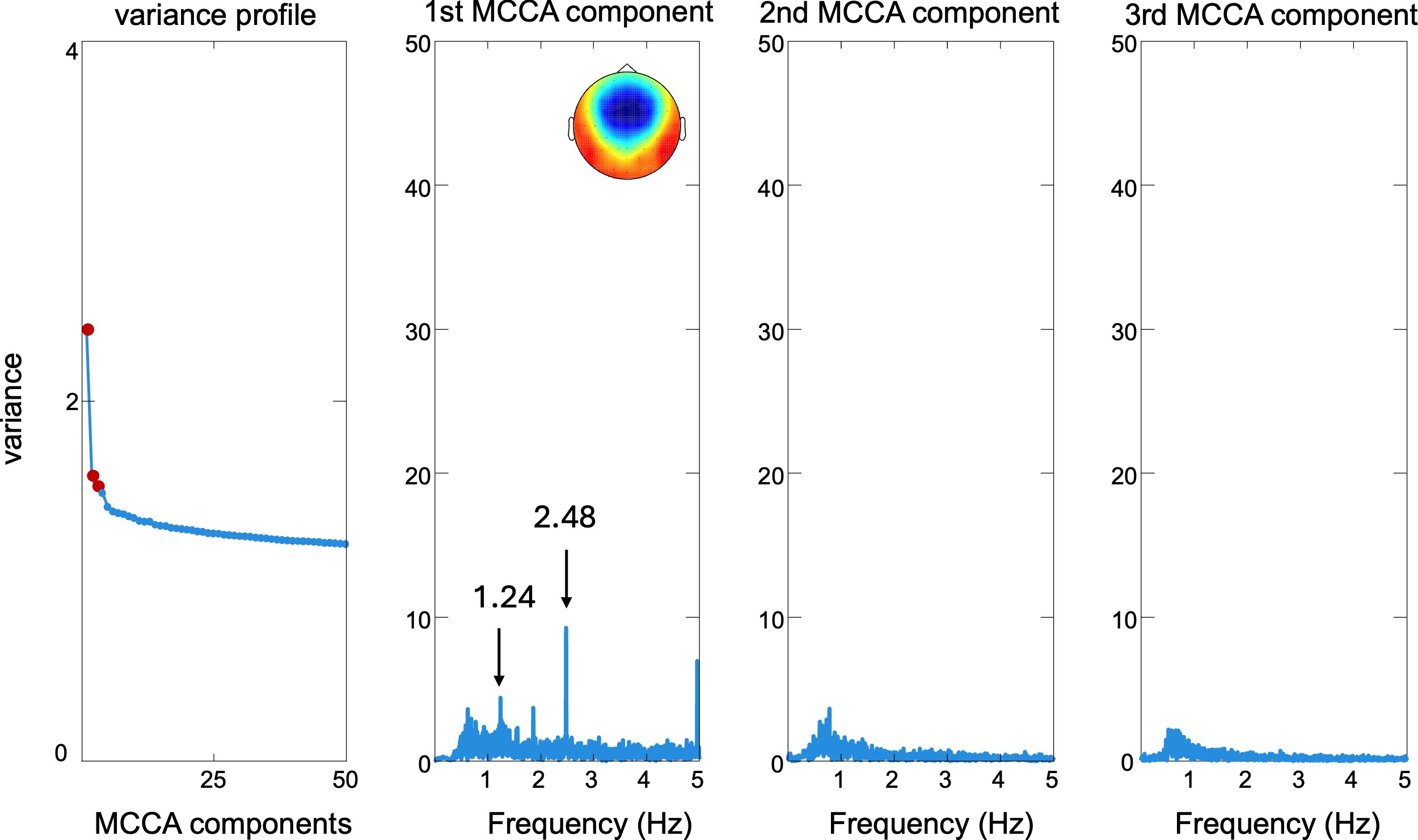
**

**S7. Processing of EEG signal – Regularisation of temporal response function (TRF)**

To prevent the model from overfitting to the specific dataset, regularisation is required. This is achieved by ridge regression, which regulates the correlation between the model weights using a hyperparameter known as the ridge parameter (λ)^5^. In the context of TRF analysis, λ, the regularisation parameter, introduces temporal smoothing during the TRF computation to ensure that the model is not overly fitted to the noise in the data. λ value is adjusted through cross-validation to maximize the correlation between the actual neural response (𝑟(𝑡, 𝑛)) and the response predicted by the model (𝑟̂(𝑡, 𝑛)). In this study, a fixed λ value of 0.1 was used, which is the minimum value to prevent overfitting while minimizing signal smoothing.

**S8.** Correlations between accuracy F-scores and subscales of Goldsmith Music Sophistication Index.

| Condition | General Index | Musical Training |
| --- | --- | --- |
| regular | r_(33)_ = 0.123, *p* = .480 | r_(33)_ = 0.523, ***p* = .001** |
| irregular | r_(33)_ = 0.031, *p* = .860 | r_(33)_ = 0.331, *p* = *.052* |
| shuffled | r_(33)_ = -0.199, *p* = .258 | r_(33)_ = -0.081, *p* = .647 |

**S9. Temporal response function over an acoustic envelope**

The temporal response function (TRF) was computed ^3^ in order to quantify the brain tracking of the acoustic envelope of the stimulus. The model explained 21% of the variance (*R^2^* = 0.21 ± 0.059).


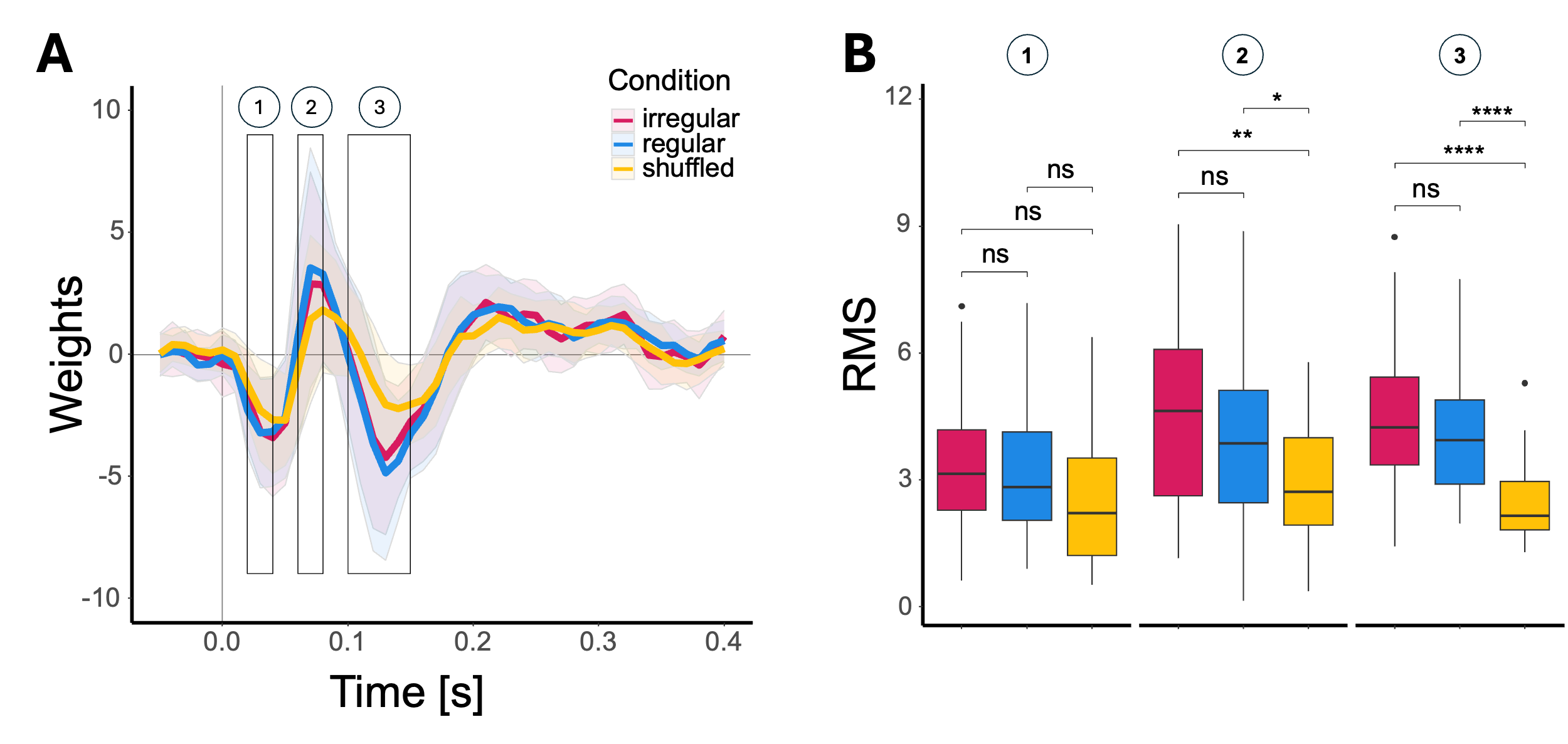
The results were compared between conditions to identify significant differences in the processing of acoustic stimuli. Cluster-based repeated measures ANOVA was performed on TRFs within the time window from 100 ms before to 400 ms after stimulation, with a significance level of *p* = .05. To assess the significance of *F* values obtained by random assignment of conditions, 1000 permutations were performed on the randomized data, and a threshold equal to 1% of the highest values obtained was applied to re-evaluate clusters and exclude chance occurrences. Later we calculated the root mean square (RMS) for each cluster across conditions and compared them using pairwise *t*-test comparison, Bonferroni corrected. The cluster-based repeated-measure ANOVA ^6^ revealed three significantly different periods between the conditions (20-40 ms, 60-80 ms and 100-150 ms). Then, we computed the root mean square (RMS) of each cluster, to obtain the magnitude of weights computed with the TRF for each cluster, for each condition. Pairwise *t*-test comparison (Bonferroni-corrected) between the RMS of the TRF for each condition within each cluster shows that the difference only occurred between regular-irregular and shuffled condition, particularly in the latest cluster. The early processing differences were rather small, as they were related to the processing of more fundamental properties of the sound ^7,8^. The difference in the last cluster, which was the largest difference, could already be caused by the presence or lack of the musical structure ^9^.

**(A**) Temporal response function (TRF) over the acoustic envelope of stimuli reveals three time windows in which there is a significant difference between conditions: 20-40 ms, 60-80 ms, and 100-150 ms after onset. (**B**) Comparison of root mean square (RMS) between conditions for three significant time windows.

Pairwise *t*-test comparisons between root mean square (RMS) values, representing the strength of the temporal response function (TRF) over acoustic envelope for the significant clusters.

| Cluster | Group 1 | Group 2 | statistic | *p* adjusted |
| --- | --- | --- | --- | --- |
| 1  20-40ms | irregular | regular | 0.0289 | .977 |
|  | irregular | shuffled | 1.87 | .188 |
|  | regular | shuffled | 1.90 | .188 |
| 2  60-80ms | irregular | regular | 1.14 | .259 |
|  | irregular | shuffled | 3.50 | **.003** |
|  | regular | shuffled | 2.41 | **.038** |
| 3  100-150ms | irregular | regular | 1.53 | .132 |
|  | irregular | shuffled | 6.31 | **< .0001** |
|  | regular | shuffled | 5.09 | **< .0001** |

**S10.** Pairwise *t*-test comparisons between conditions for the beat and half beat tracking by the brain obtained with cerebral-acoustic coherence (Cacoh).

| Frequency | Group 1 | Group 2 | statistic | *p* adjusted |
| --- | --- | --- | --- | --- |
| beat  (1.24 Hz) | irregular | regular | -0.817 | .995 |
|  | irregular | shuffled | 6.88 | **< .0001** |
|  | regular | shuffled | 8.99 | **< .0001** |
| half beat  (2.48 Hz) | irregular | regular | -2.17 | .123 |
|  | irregular | shuffled | -3.96 | **< .0001** |
|  | regular | shuffled | -1.91 | .159 |

**References**

1. Grahn, J. A. & Brett, M. Rhythm and Beat Perception in Motor Areas of the Brain. *Journal of Cognitive Neuroscience* **19**, 893–906 (2007).

2. De Cheveigné, A. *et al.* Multiway canonical correlation analysis of brain data. *NeuroImage* **186**, 728–740 (2019).

3. Crosse, M. J., Di Liberto, G. M., Bednar, A. & Lalor, E. C. The Multivariate Temporal Response Function (mTRF) Toolbox: A MATLAB Toolbox for Relating Neural Signals to Continuous Stimuli. *Front. Hum. Neurosci.* **10**, (2016).

4. Hardoon, D. R., Szedmak, S. & Shawe-Taylor, J. Canonical Correlation Analysis: An Overview with Application to Learning Methods. *Neural Computation* **16**, 2639–2664 (2004).

5. Fathi, E. & Maleki Shoja, B. Deep Neural Networks for Natural Language Processing. in *Handbook of Statistics* vol. 38 229–316 (Elsevier, 2018).

6. Oostenveld, R., Fries, P., Maris, E. & Schoffelen, J.-M. FieldTrip: Open Source Software for Advanced Analysis of MEG, EEG, and Invasive Electrophysiological Data. *Computational Intelligence and Neuroscience* **2011**, 1–9 (2011).

7. Wang, K. & Shamma, S. A. Auditory analysis of spectro-temporal information in acoustic signals. *IEEE Eng. Med. Biol. Mag.* **14**, 186–194 (1995).

8. Nelken, I. Neurons and objects: the case of auditory cortex. *Front. Neurosci.* **2**, 107–114 (2008).

9. Teng, X., Larrouy-Maestri, P. & Poeppel, D. Segmenting and Predicting Musical Phrase Structure Exploits Neural Gain Modulation and Phase Precession. *J. Neurosci.* e1331232024 (2024) doi:10.1523/JNEUROSCI.1331-23.2024.
